# Biodegradation Potential of *Bacillus* sp. PAH-2 on PAHs for Oil-Contaminated Seawater

**DOI:** 10.3390/molecules27030687

**Published:** 2022-01-21

**Authors:** Xianghui Kong, Ranran Dong, Thomas King, Feifei Chen, Haoshuai Li

**Affiliations:** 1 Fisheries College, Ocean University of China, Qingdao 266003, China; xianghuikong.ouc@gmail.com; 2 Frontiers Science Center for Deep Ocean Multispheres and Earth System, Key Laboratory of Marine Chemistry Theory and Technology, Ministry of Education, Ocean University of China, Qingdao 266100, China; dongranran@stu.ouc.edu.cn (R.D.); Chenfeifly199101@163.com (F.C.); 3 College of Chemistry and Chemical Engineering, Ocean University of China, Qingdao 266100, China; 4 Department of Fisheries and Oceans, Bedford Institute of Oceanography, Dartmouth, NS B2Y 4A2, Canada; tom.king@dfo-mpo.gc.ca

**Keywords:** aromatic components, crude oil, bioremediation, PAHs, oil-contaminated seawater

## Abstract

Microbial degradation is a useful tool for inhibiting or preventing polycyclic aromatic hydrocarbons (PAHs) widely distributed in marine environment after oil spill accidents. This study aimed to evaluate the potential and diversity of bacteria Bacillus sp. PAH-2 on Benzo (a) anthracene (BaA), Pyrene (Pyr), and Benzo (a) pyrene (BaP), their composite system, aromatic components system, and crude oil. The seven-day degradation rates against BaA, Pyr, and BaP were 20.6%, 12.83%, and 17.49%, respectively. Further degradation study of aromatic components demonstrated PAH-2 had a high degradation rate of substances with poor stability of molecular structure. In addition, the degradation of PAHs in crude oil suggested PAH-2 not only made good use of PAHs in such a more complex structure of pollutants but the saturated hydrocarbons in the crude oil also showed a good application potential.

## 1. Introduction

Over the past few decades, several serious oil spill accidents have occurred, which caused serious pollution to the marine environment. For instance, the Deepwater Horizon oil spill occurred in the Gulf of Mexico in 2010 [1,2] has been estimated that about 4.1 million barrels of oil spilled into the open ocean [3]. The negative ecological environment impacts of oil spills are not only due to the toxic effects of petroleum itself but also result of secondary pollution and indirect damages such as hydrological effects [4]. Moreover, it is worth noting that approximately 3.9% of PAHs by weight were released during the Deepwater Horizon oil spill accident [4]. Studies have shown that the concentration of naphthalene and helium near the oil spill accident in the surface seawater is 0.13 and 0.34 mg/L, respectively, while the concentration of total PAHs in the surface water of the marine environment is normally ng/L [5].

PAHs are representative pollutants in petroleum, widely distributed in the environment [6,7]. It is reported that PAHs are considered to be the most toxic and abundant organic carcinogens [8]. PAH is readily absorbed by suspended particles in water. In addition, it is prone to deposition due to its hydrophobicity which makes it difficult to eliminate in the environment [9]. Highly toxic, mutagenic, and carcinogenic effects caused by some PAHs have an interaction on microorganisms [10,11]. PAHs are classified as B2 carcinogenic substances: Once these substances came into the human body, it is easily purveyed to the human nervous system and hematopoietic system which affects human organ lesions [12,13]. Meanwhile, PAHs also have the biological characteristics of bioaccumulation, biomagnification, and persistent toxicity. Their cumulative accumulation of toxicities through the food chain poses a serious threat to the biological population and human health in the aquatic environment and leads to ecosystem degeneration and degradation [14]. Moreover, as toxic compounds, PAHs may also have a serious impact on the survival of phytoplankton, zooplankton, marine shellfish, and fish [15]. In addition to the oil spill problem, PAHs in seawater can be produced in many activities of people such as wastewater [16]. Therefore, the PAHs remediation in the polluted ocean environments has become a critical issue and a global challenge [17]. Marine oil spills are mainly treated by various combinations of physical methods, chemical methods, and microbial degradation [18,19,20], and many new efficient methods have come out in recent years, such as photocatalyst and electro-activated oxidants, etc. [21]. Compared to other methods, microbial degradation has become an ideal method to remove the PAHs because of its economical, high efficiency, and free of secondary pollution [22].

To make the microbial remediation applications feasible, screening out bacteria which have highly efficient degradation rates is the first work. More than 70 genera and 200 different kinds of microbes have been discovered which could degrade hydrocarbons, most of them bacteria and fungi [23]. Microbial degradation of PAHs is mainly based on bacteria and fungi. Bacteria catalyze the oxygenation of PAHs by generating dual oxygenases, while fungi can secrete lignin-degrading enzymes or monooxygenases to oxidize PAHs [24]. In recent years, it was found that the following microorganisms can degrade PAHs: *Rhodococcus*, *Micrococcus*, *Cyanobacteria*, *Nocardia*, *Corynebacterium*, *Aeromonas*, *Flavobacterium*, *Pseudomonas*, *Beijerinckia*, *Mycobacterium*, *Bacillus*, and *Vibrio* [25]. In addition, white-rot fungi were found to have a strong ability to degrade PAHs [26].

The core of PAHs degradation research is the degradation mechanism and the screening of highly efficient degrading bacteria. Therefore, research on this area has become very popular and make encouraging progress in recent years. Two microbial pathways of PAHs degradation have been found: 1. PAHs as carbonates; and 2. the degradation of PAHs and other co-metabolites [26]. Among them, the co-metabolism of microorganisms plays a significant role in the complete decomposition or mineralization of refractory PAHs [27]. The biodegradation of PAHs is affected by a series of enzymatic reactions, which play critical roles in biodegradation [28].

BaA, Pyr, and BaP exist in the environment, and have the biodegradation of 4,4,5 rings and similar structures, while their corresponding toxic effects vary greatly. The study of their degradation differences is helpful to understand the similarities and differences in the degradation of PAHs with similar structures and ring numbers, so as to enrich the degradation theory of PAHs. The objectives of this study include: 1. to screen the degrading bacteria with a high degradation rate of PAHs (BaA, Pyr, and BaP) and to analyze the molecular identification of bacteria by amplifying and sequencing the 16S rRNA gene; 2. to obtain the optimal degradation conditions including temperature, pH, concentration (different PAHs concentrations), and substrate concentration (initial PAHs concentration); 3. to research the relationship between degradation rule and the hydrophobicity of the bacteria by 35 days’ comparison degradation experiments; and 4. Particularly, to research the degradation against composite PAHs, pure aromatic components, and crude oil.

## 2. Materials and Methods

### 2.1. Materials

#### 2.1.1. Chemicals

BaA (purity P98%), Pyr (purity P97%), and BaP (purity P96%) were obtained from Aladdin Chemistry Co., Ltd. (Shanghai, China). Crude oil was obtained from Shengli oilfield (Dongying, China). The pertinent characteristics of the crude oil (viscosity, density, and freezing point) can be found in our previous work [20].

#### 2.1.2. Solutions and Media

The enrichment medium was composed of 0.5 g of beef extract, 1.0 g of peptone, and 0.5 g of NaCl. The beef extract peptone medium included 0.5 g of beef extract, 1.0 g of peptone, 0.5 g of NaCl, and 15–25 g of agar (solid). The mineral salts medium contained 5 g of NaCl, 1 g of (NH_4_)_2_SO_4_; 0.25 g of MgSO_4_·7H_2_O; 2 g of NaNO_3_; 10 g of K_2_HPO_4_·3H_2_O; and 4 g of KH_2_PO_4_. The domestication medium consisted of 5 g of NaCl; 1 g of (NH_4_)_2_SO_4_; 0.25 g of MgSO_4_·7H_2_O; 2 g of NaNO_3_; 10 g of K_2_HPO_4_·3H_2_O; and 4 g of KH_2_PO_4_, with BaA, Pyr, and BaP as the sole carbon source, respectively. All media were adjusted to pH 7.2–7.4 with hydrochloric acid or sodium hydroxide solution and autoclaved at 121 °C for 20 min before use.

PAH solution was prepared with 500 mg of BaA, Pyr, and BaP dissolved with acetone in a 100-mL volumetric flask, respectively. One mL of solution was added to a 99-mL Erlenmeyer flask with the mineral salt medium. The acetone was completely volatilized by shaking at 40 °C and 150 rpm for 8 h. The solutions were then irradiated by UV lamp for 2 h for sterilization. Finally, the concentration of each PAH (BaA, Pyr, and BaP) was 50 mg/L.

### 2.2. Bacteria Isolation with the High Efficient Degradation Rate

The bacteria were screened from sludge contaminated by petroleum hydrocarbons of Shengli Oil Field. The 1 g of sludge was placed into the domestication medium with the PAHs, and cultivated in a gas bath with a thermostatic oscillator at 30 °C, 120 rpm for seven days. One mL of enrichment bacterium liquid was then added to fresh domesticated culture medium and continued to be cultivated under the same conditions, recycled three times. The bacteria inoculated to the beef extract peptone medium after 21 days were developed in a biochemical incubator 25 °C for two days. Three bacterial species were obtained and stored in a glycerin tube in a refrigerator at −80 °C for future research use. After enrichment, the bacterial consortia were serial diluted to 10^8^ and spread on an agar plate with the same composition of the enrichment medium.

The appropriate amount of the three bacteria were added to the enrichment medium, cultivated for 48 h, and inoculated of the bacterium liquid were inoculated to mineral salt medium with BaA, Pyr, and BaP, respectively, in culture flasks (the concentration of the three PAHs were all 50 mg/L). The three PAHs in the medium were all degraded for seven days. The compounds were then extracted by n-hexane (AR) and diluted with n-hexane to 50 mL, then the concentrations were measured by UV-vis spectrophotometer to determine the absorbance change of PAHs during degradation at wavelengths of 287, 240, and 296 nm, respectively, using pure n-hexane as a blank control. Each experiment was repeated three times. The resulting degradation rates were then determined from standard curve equations prepared from dilutions of the standard compounds [29]. From these measurements, the highly efficient degradation rates of the bacteria were obtained.

Finally, 5 mL of the enriched bacterium liquid was inoculated to the new enrichment medium, measured by UV-Vis spectrophotometer at 600 nm every hour from 0 h, with the fresh enrichment medium used as a blank control. The growth curve of the bacteria was then obtained after 30 h. The plate count method was used to study the relationship between the number of bacteria and the absorbance value.

### 2.3. Identification of the Optimum Selected PAHs Degradation Bacteria

Through a series of experiments in Section 2.2, we screened out the optimum degradation bacteria. These selected isolated bacteria were identified by standard biochemical tests (Gram staining, spore staining, and oxidation/fermentation test), Methyl red experiment, Voges–Proskauer, indole, and the hydrolysis of cellulose [30]. The bioassay of bacteria was performed by the Beijing Genomics Institute (BGI). Isolated strains were classified and identified by 16S rRNA. Total DNA was extracted by CTAB method [31,32]. The sequence is analyzed and correlated with the system according to the scheme [33]. The phylogenetic tree was plotted by MEGA5 software with the neighbor-joining method.

### 2.4. External Factors Affecting Degradation of PAH (BaA, Pyr and BaP)

#### 2.4.1. Contaminant Concentration

Five mL of bacteria solution in stable growth period was taken and inoculated into a 95-mL solution, each containing different PAHs (BaA, Pyr, and BaP), respectively. The above experiment was repeated three times. The mixtures were then cultured at a pH = 7 for seven days while mixing at 120 rpm in a shaker flask (temperature, 30 °C, and salinity, 5 g/L). The specific concentrations were set at 30, 40, 50, 60, 70 mg/L, respectively.

#### 2.4.2. Temperature

**Figure 1:**
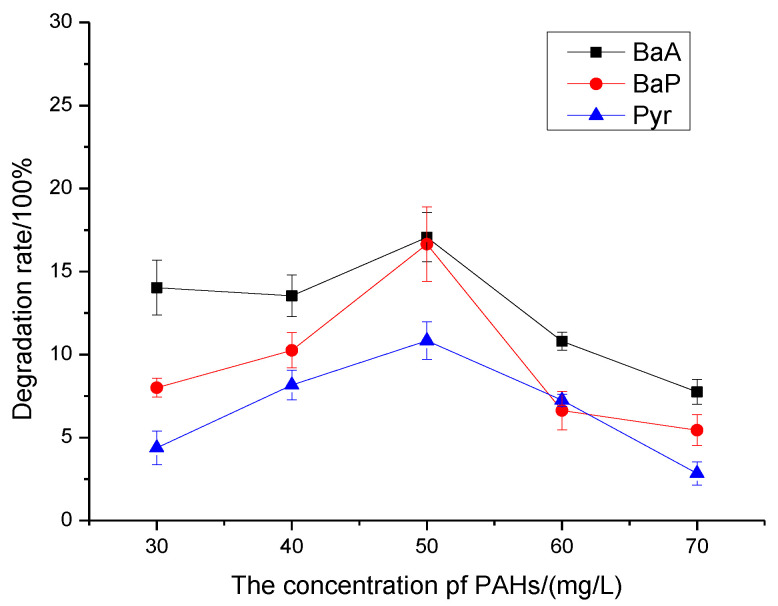
PAH-2 degradation rate of BaA, Pyr and BaP under different substrate concentration.

As shown in Figure 1, BaA had the maximum degradation rate when the substrate concentration was 50 mg/L, so the reactant concentration of the rest of the experiments was set at 50 mg/L.

Five mL of bacteria solution in stable growth period was taken and inoculated into a 95-mL solution, each containing 50 mg/L of PAHs (BaA, Pyr, and BaP), respectively. The above experiment was repeated three times. The mixtures were then cultured at a specific temperature for seven days, while mixing at 120 rpm in a shaker flask (pH, 7 and salinity, 5 g/L). The specific temperatures were set at 20, 25, 30, 35 °C and 40 °C, respectively.

#### 2.4.3. pH

Five mL of bacteria solution in stable growth period was taken and inoculated into a 95-mL solution, each containing 50 mg/L of PAHs (BaA, Pyr, and BaP), respectively. The above experiment was repeated three times. The mixtures were then cultured at a specific pH for seven days while mixing at 120 rpm in a shaker flask (temperature, 30 °C and salinity, 5 g/L). The specific pH values were: 6.0, 6.5, 7.0, 7.5 and 8.0, respectively.

#### 2.4.4. NaCl Concentration

Five mL of bacteria solution in stable growth period was taken and inoculated into a 95-mL solution, each containing 50 mg/L of PAHs (BaA, Pyr, and BaP), respectively. The above experiment was repeated three times. The mixtures were then cultured at a specific NaCl concentration for seven days, while mixing at 120 rpm in a shaker flask (temperature, 30 °C and pH, 7). The specific NaCl concentrations were set as: 2, 5, 7, 10, and 15 mg/L, respectively.

#### 2.4.5. PAHs Concentration

Five mL of bacteria solution in stable growth period was taken and inoculated into 95-mL solutions of varied PAH concentrations, each containing 30, 40, 50, 60, or 70 mg/L PAHs (BaA, Pyr, and BaP), respectively. The above experiment was repeated three times. The mixtures of specific initial PAH concentrations were cultured for seven days, mixing at 120 rpm in a shaker flask (temperature 30 °C, pH 7, and salinity 5 g/L).

For each experiment mentioned above, the medium without PAHs served as a control group and three parallel samples were used in each test. The amounts of the PAHs remaining in the shaker flask were measured after seven days’ of culturing and the degradation rate of each shaker flask was calculated to obtain a temperature suitable for the degradation of PAH-2.

### 2.5. Degradation Process of Three Single PAHs

#### 2.5.1. The Growth of Bacteria in the Degradation with Time

After 3, 5, 10, 15, 20, 25, 30, and 35 days, the bacterium liquid was obtained after extraction. The bacterial numbers were obtained by UV-vis spectrophotometer through the relationship between the number of bacteria and the corresponding absorbance value.

#### 2.5.2. Surface Hydrophobicity of Bacteria with Time

The surface hydrophobicity of the bacteria was measured by contact angle meter at 10, 20, and 30 days. First, the bacterium liquid (after extracting PAHs) was filtered through a microporous membrane having a 0.22 μm aperture, and the membrane should be naturally dried and a smooth surface maintained. The membrane was then cut into a square of 1 × 1 cm, set it into a sample pool with n-hexane as the oil phase, and set on a platform in an appropriate place in the pool for taking photographs. The needle of a syringe filled with MilliQ water was then placed into the organic phase liquid level and the syringe plunger gently pressed in order to put a drop of MilliQ water on the bacterial membrane. A photograph was taken at the moment of contact, and the size of the contact angle between the droplet and the membrane represents the hydrophobicity of the bacterium.

### 2.6. Quantification of Composite PAHs by GC-FID Analysis

The composite PAHs were quantitatively analyzed with a GC equipped with a DB-5 column (30 m × 0.25 mm × 0.25 μm, J&W Scientific, Folsom, CA, USA), an autosampler (AOC 20i), and a FID. The method for this PAHs biodegradation study and the collection of oil contaminated seawater was used the described in [34]. All GCMS samples were in duplicate for testing and to ensure repeatability of data.

## 3. Results and Discussion

### 3.1. The Effect of Contaminant Concentration

PAHs degrading bacteria have different degradation performance for different pollutants, and different pollutant substrate concentrations also have an impact on the growth of microorganisms, thus affecting the degradation rate of pollutants. If the concentration of pollutants is too high, it causes great toxic effect on microorganism and inhibit the growth of microorganism. If the concentration of pollutants is too low, it cannot provide sufficient carbon source for the growth of microorganisms, which is not conducive to the growth of microorganisms, and the degradation of pollutants was unable to play a maximum role. Therefore, it is very important to explore the environment of pollutant concentration to which microorganisms adapt.

As can be seen from Figure 1, within the range of experimental concentration, PAHs degradation by PAH-2 basically increased first and then decreased with the increase of substrate concentration, and the degradation rate reached the maximum when substrate concentration was 50 mg/L. The maximum degradation rates of BaA, BaP and Pyr were 17.06%, 16.65%, and 10.84%, respectively. It can be seen that when the concentration is less than 50 mg/L, the degradation of BaA and BaP is considerable. When the concentration is greater than 50 mg/L, the degradation rate of the three kinds of PAHs will decrease to a large extent with the increase of the concentration. However, in practical application, we maximize the degradation ability of microorganisms. In the subsequent studies, 50 mg/L was selected as the optimal concentration of contamination substrate. As can be seen from the figure above, the degree of biodegradation of PAH-2 was BaA ≤ BaP < Pyr, that is, PAH-2 had a lower degradation rate for Pyr with relatively stable structure, and a slightly higher degradation rate for BaA and BaP with larger molecular weight and stronger hydrophobicity.

### 3.2. Degradation Efficiency of PAHs Degrading Bacteria

**Figure 2:**
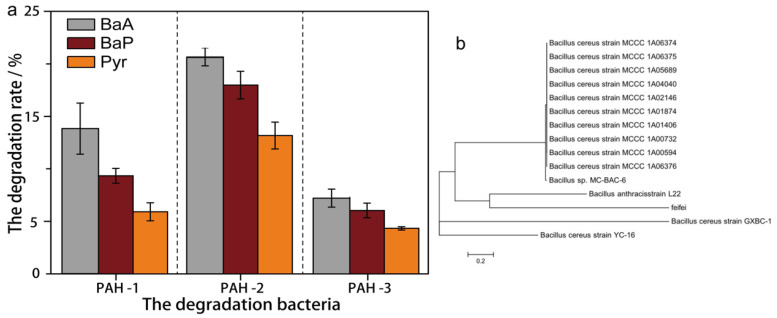
(**a**) The degradation of BaA, Pyr, and BaP by PAH degradation bacteria after seven days. Each degradation experiment was repeated three times to ensure repeatability and accuracy of the data. (**b**) Phylogenetic tree of PAH degrading Bacillus PAH-2.

The biodegradation results for BaA, Pyr, and BaP after the biodegradation period are presented in Figure 2a. The three strains were PAH-1, PAH-2, and PAH-3, respectively. The biodegradability of them on BaA, Pyr, and BaP followed the order: PAH-2 > PAH-1 > PAH-3. Over seven days, the degradation efficiencies of the three strains for BaA were 13.80%, 20.60%, and 7.21%, respectively; the degradation efficiencies for Pyr were 5.75%, 12.83%, and 4.21%, respectively; and the degradation efficiencies on BaP were 9.08%, 17.49%, and 5.88%, respectively. The PAH-3 strain can also utilize petroleum hydrocarbons as carbon sources; however, its metabolic rate was significantly slower than those of either PAH-2 or PAH-1, with 41.7% of total n-alkanes removed. PAH-2 showed significantly higher degradation ability on BaA, Pyr, and BaP than the other two strains.

### 3.3. Bioassay of PAH-2 Bacteria

The 16S rRNA gene was amplified and sequenced, and compared with the known 16S rRNA sequence database for bacterial molecular identification. We selected the most suitable PAHs degradation bacteria through degradation experiments above, so we just tested the phylogenetic tree of PAH-2. The phylogenetic of the optimal degradation strain is shown in Figure 2b. The feifei was represented by PAH-2 bacteria. The strains selected to construct the phylogenetic tree were more than 99% similar to bacillus in nucleotide sequence. Sequence analysis of 16S rRNA gene and phylogenetic analysis of BLAST sequence was performed to confirm that the bacteria belonged to bacillus. In addition, the morphology, physiological, and biochemical characteristics of bacteria can be found in Table S1.

### 3.4. Effect of Environmental Factors on the PAHs Degradation by PAH-2

**Figure 3:**
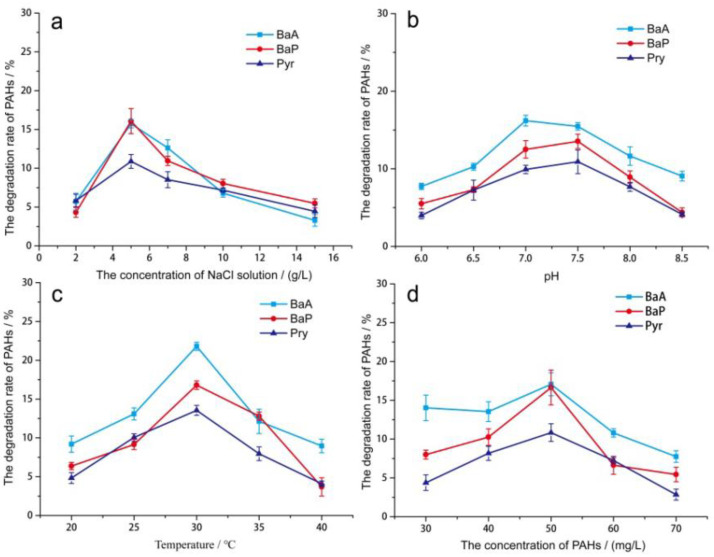
PAH-2 degradation rate of BaA, BaP, and Pyr at different values of: temperature (**a**), pH (**b**), NaCl concentration (**c**), PAH concentration (**d**). In the figure, the red lines represented the degradation, and the blue lines represented the CFU/mL. Each degradation experiment was repeated three times to ensure repeatability and accuracy of the data.

The change of temperature can affect the activity of bacterial decomposition enzymes and the analysis and distribution coefficients of pollutants [26], to control the growth and degradation of microorganisms. Figure 3a shows that the bacterial PAH-2 degradation of BaA, Pyr, and BaP constantly increased and then decreased over the experimental temperature range. The optimal degradation temperature was 30 °C. At the optimal degradation temperature, the biodegradability degree of the three PAHs was BaA > BaP > Pyr. In the pH range, BaA, Pyr, and BaP all had different degrees of degradation (Figure 3b). However, they exhibited the same trend and maximum degradation rate (pH, 7.0~7.5). These results are consistent with those of other researchers [35]. Inorganic salts play an important role in the process of enzyme reactions, maintaining the equilibrium of cell membranes and regulating the osmotic pressure [16,36]. Proper salinity can optimize the physiological state of microorganisms and make the enzymatic reactions play the most important role. In this study, the bacterial PAH-2 degradation of BaA, BaP, and Pyr had the optimal NaCl concentration (5 g/L), and their corresponding biodegradation rates were 15.74%, 16.07%, and 10.88%, respectively (Figure 3c). Compared with their biodegradability, we found BaA > BaP > Pyr at the optimal NaCl concentration. In addition, the PAHs concentration effect on degradation was investigated as shown in Figure 3d. The PAH-2 degradation of BaA, BaP, and Pyr all basically exhibited a downward trend after an initial rising trend as the substrate concentration increases. BaA, BaP, and Pyr ‘s degradation rate reached its maximum when the substrate concentration was 50 mg/L. The maximum degradation rates were 17.06%, 16.65%, and 10.84%, respectively. The biodegradation abilities of the three were in the order BaA > BaP > Pyr. From Figure 3, no matter what kind of environmental variable was examined, the PAH-2 degradation of BaA, BaP, and Pyr obeyed an objective law or order which was BaA > BaP > Pyr. PAH-2 degradation is relatively tricky for Pyr, which has relatively stable structure. For BaA and BaP, both with larger molecular weights and being strongly hydrophobic, PAH-2 degradation is relatively easy.

### 3.5. Degradation Process of Three PAHs by PAH-2

#### 3.5.1. Degradation Rate of Single PAHs with Time

**Figure 4:**
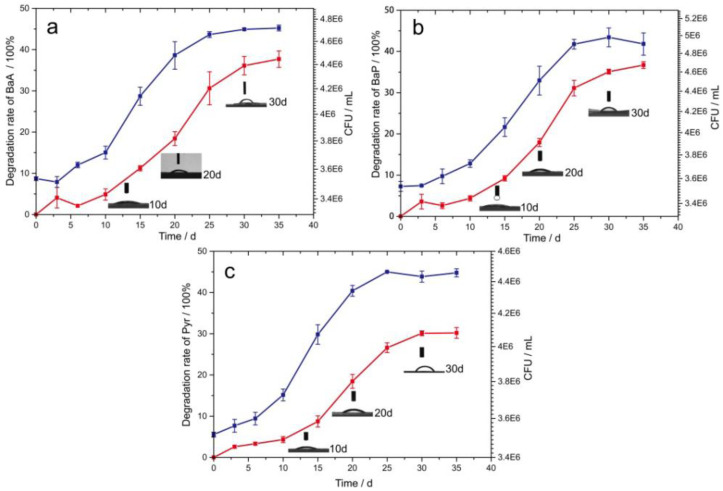
PAH-2 degradation rates of (**a**) BaA, (**b**) BaP, and (**c**) Pyr and the bacteria number during the degradation process changes with time and the black inserts (10, 20, 30 d) represent the bacterial hydrophobicity during degradation. Each experiment was repeated three times to ensure repeatability and accuracy of the data.

There were many similarities between the degradation of the three-single polycyclic aromatic hydrocarbons and the change of the bacterial population. The degradation rates of BaA, Pyr, and BaP were low, and the bacterial PAH-2 growth was also very slow within 10 days. During the initial stage of bacterial degradation, the bacteria were in an environment-adapted stage [37]. The degradation rate and the number of bacteria showed BaA > BaP > Pyr during the same period. The toxicity of PAHs affects the time required for the bacteria to adapt to the environment [38], and the degradative capacity decreases as the toxicity increases. After a period, the bacteria adapt to the toxicity of the pollutants. The bacteria were then more favorable about the degradation of the three PAHs, showing a faster degradation rate and a corresponding increase in the number of bacteria. Due to the gradual accumulation of toxins in the bacteria, after 20 days of degradation, the number of bacteria increased more slowly, and the degradation rate of PAHs did not increase much after 25 days (Figure 4).

#### 3.5.2. Changes in Bacterial Hydrophobicity during Degradation

Bacterial hydrophobicity has been used to investigate the adhesion of microorganisms to many surfaces, including the co-adhesion of bacteria and oil droplet [39,40]. Figure 4 also shows that the changes of bacterial hydrophobicity during the degradation of the three PAHs were similar. The hydrophobicity of the bacteria gradually increased, and the lipophilicity of the bacteria accordingly gradually increased [41]. At 10 d, the hydrophobicity was very small. At this time, the bacteria were in the stage of adapting to the environment, and the degree of degradation of the PAHs was small. The hydrophobicity further increased after 20 d, when the bacteria had adapted to the toxic environment caused by the organic pollutants [42]. The PAHs also degraded to some extent. By the 30th day, the contact angles of the bacterial filters were already large. At this time, the bacteria have been well adapted to the environment, and the utilization of PAHs was also higher.

### 3.6. Degradation of PAH Mixtures, Aromatic Components, and Crude Oil by PAH-2

#### 3.6.1. Mixed PAHs Degradation Efficiency

**Figure 5:**
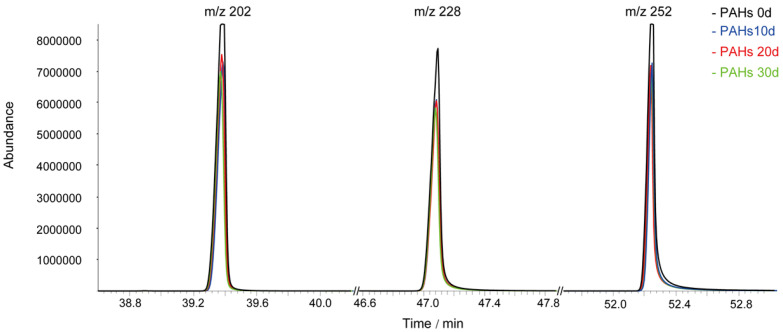
The degradation effect of PAH degrading Bacillus PAH-2 in composite PAHs. The *m*/*z* 202, *m*/*z* 228 and *m*/*z* 252 represented BaA, Pyr, and Bap, respectively.

When the three PAHs (BaA, Pyr, and BaP) exist simultaneously in the same environment, bacteria may preferentially degrade one or more of these substances according to their degradation substrate preferences [43]. The PAH degradation ability of PAH-2 bacteria to degrade BaA, Pyr, and BaP is shown using GC-FID analysis in Figure 5. The *m*/*z* 202, *m*/*z* 228, and *m*/*z* 252 represented BaA, Pyr, and Bap, respectively. From a macroscopic point of view, the PAH-2 degradation of the three substances gradually increased over time. The degradation rates of PAH-2 for BaA, Pyr, and BaP are about 45%, 40%, and 45%, respectively. The degradation effect was not obvious at 20 days; we can see that the degradation rate was relatively slow, and in the 20- to 30-day span, the impacts of PAH-2 on the degradation of the three PAHs were considerable and showed a higher rate of degradation. It is worth noting that PAH-2 preferentially degraded Pyr in the environment for 30 days. Overall, Pyr was the least degraded compound, with only BaA and BaP showing remarkable significances after 30 days. It can be surmised that the bacterial degradation rates of molecules with good molecular stability are relatively low and are not affected by the number of rings and hydrophobicity [44].

Some scholars have determined that the more complex structure of the PAHs, the lower the bacterial degradation rate [45]. These findings are consistent with previous research that degradation rates generally decrease with increasing molecular weight [46,47]. This phenomenon has been attributed to the compound’s water solubility. Specifically, low molecular weight PAHs are more soluble and bioavailable as they were first used by microbes [48]. Another explanation for this phenomenon is competitive inhibition, which is common in mixed matrix cultures. Under competitive inhibition, more soluble PAHs were inhibited by enzymes used to degrade high molecular weight PAHs [49,50].

#### 3.6.2. Degradation Efficiency of Pure Aromatic Components

**Figure 6:**
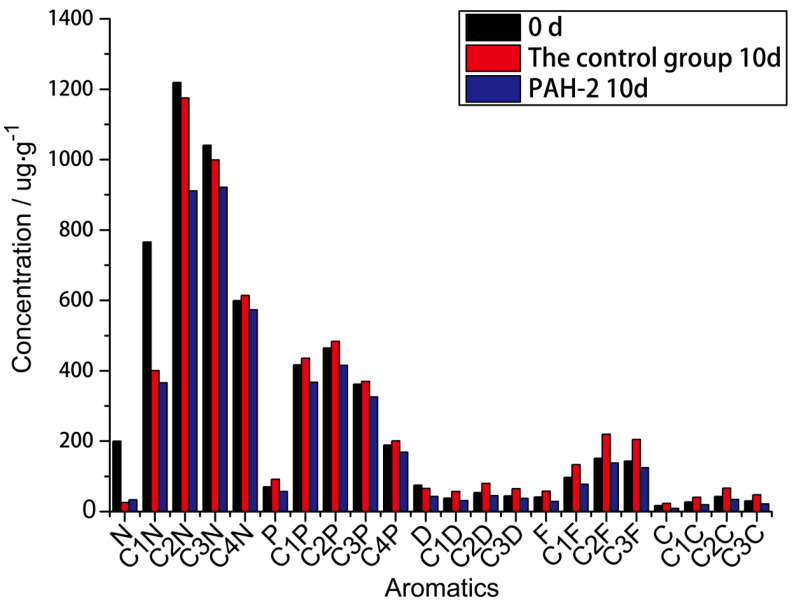
The degradation effect of PAH degrading bacillus PAH-2 in aromatic components across 10 days through GS-MS data.

It was found that after 10 days of degradation with PAH-2, most of the aromatic components of the material had been degraded, indicating that the bacteria had a high utilization rate of this material within 10 days. From Figure 6, we can see the degradation effect of PAH degrading bacillus PAH-2 in aromatic components across 10 days through GS-MS data. The resulting algorithm was detailed in [29]. The entire characteristics of the spectrum may be mostly saturated hydrocarbon components, and the degradation rate of the relatively stable naphthalene is small, not because of the ring structure of phenanthrene, but mainly due to the powerful hydrophobic effects.

#### 3.6.3. The Efficiency of PAH-2 on the Degradation of Crude Oil

**Figure 7:**
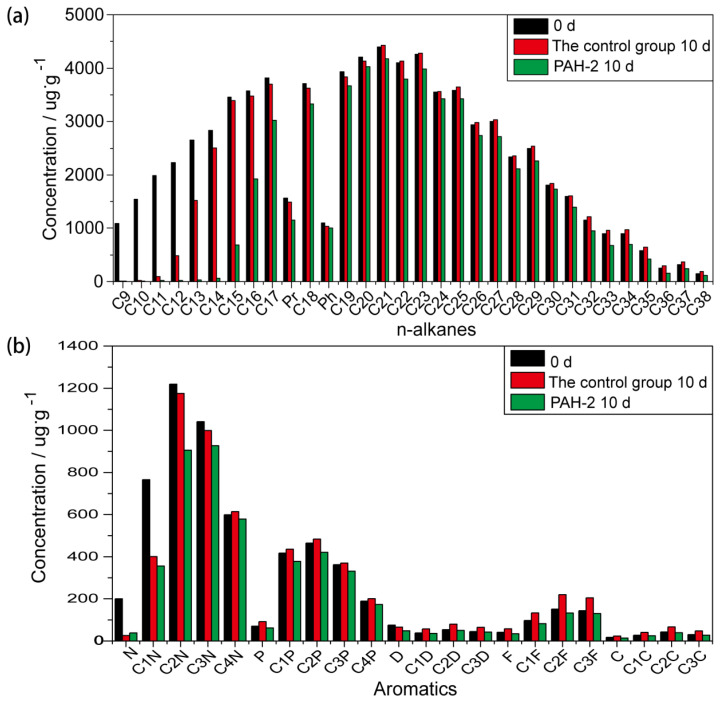
The percentage degradation of n-alkanes and aromatics in the crude oil by PAH-2 for 10 days. (**a**) the degradation in the chain length of hydrocarbon. (**b**) the degradation in aromatic components.

Using GC-MS, crude oil utilization by PAH-2 for 10 days was studied. The efficiency of PAH-2 to degrade various fractions of crude oil are shown in Figure 7. Degradation rate after 10 days of incubation was over 80% for C18~C38 and 70% for aromatics in crude oil. Figure 7a shows the rate of degradation decreased with an increase in the chain length of hydrocarbon [51]. Moreover, many short-chain hydrocarbons have been completely degraded. Aromatic components are high molecular weight and strong polar components that are difficult to degrade in crude oil (Figure 7b), which contributes to the difficulty of oil recovery [52]. Compared with other reported strains [53,54,55,56], PAH-2 strain can use a wider range of crude oil components, including aliphatic hydrocarbons (C9~C38), showing the superiority of PAH-2 strain in degrading heavy crude oil.

## 4. Conclusions

In this study, degrading bacteria (PAH-2) were screened and optimized, with a high degradation rate against BaA (20.6%), Pyr (12.83%), and BaP (17.49%) for 7 days. Furthermore, pure aromatic components degradation found PAH-2 had a high degradation rate of substances with poor stability of molecular structure. In addition, from the 10-day study of crude oil degradation, it is evident that PAH-2 not only made good use of the PAHs in such a more complex structure of pollutants, but the saturated hydrocarbons in the crude oil also showed a good potential. In conclusion, through this study, PAH-2 has an excellent power in degradation, and this bacterium will provide an extra way of controlling or preventing PAHs in oil spill accidents or other forms of pollution.

## Supplementary Materials

The following supporting information can be downloaded at, Figure S1: The standard curve of Benzo (a) anthracene (a), Pyrene (b) and Benzo (a) pyrene (c) concentration; Figure S2: The growth curve of the bacteria; Figure S3: The standard curve of the number of bacteria and the absorbance value; Table S1: Morphological, physiological and biochemical properties of bacteria from oil-contaminated sludge.

## Sample Availability

Samples of the compounds are available from the authors.
