# Supplementary material for: Biodegradation Potential of Bacillus sp. PAH-2 on PAHs for Oil-Contaminated Seawater
Source: Molecules. 2022 Jan 21;27(3):687. doi: 10.3390/molecules27030687 (PMC8839208; doi:10.3390/molecules27030687)
Supplement: Supplementary file 1 [file molecules-27-00687-s001.zip › molecules-1533065-supplementary.pdf]

Biodegradation Potential of *Bacillus* sp. PAH-2 on PAHs for Oil-Contaminated Seawater

Xianghui Kong [1], Ranran Dong [2,3], Thomas King [4], Feifei Chen [2,3] and Haoshuai Li [2,3,*]

1 Fisheries College, Ocean University of China, Qingdao 266003, China; xianghuikong.ouc@gmail.com

2 Frontiers Science Center for Deep Ocean Multispheres and Earth System, and Key Laboratory of Marine Chemistry Theory and Technology, Ministry of Education, Ocean University of China, Qingdao 266100, China; Chenfeifly199101@163.com

3 College of Chemistry and Chemical Engineering, Ocean University of China, Qingdao 266100, China; dongranran@stu.ouc.edu.cn

4 Department of Fisheries and Oceans, Bedford Institute of Oceanography, Dartmouth, NS B2Y 4A2, Canada; tom.king@dfo-mpo.gc.ca

* Correspondence: lihaoshuai@ouc.edu.cn

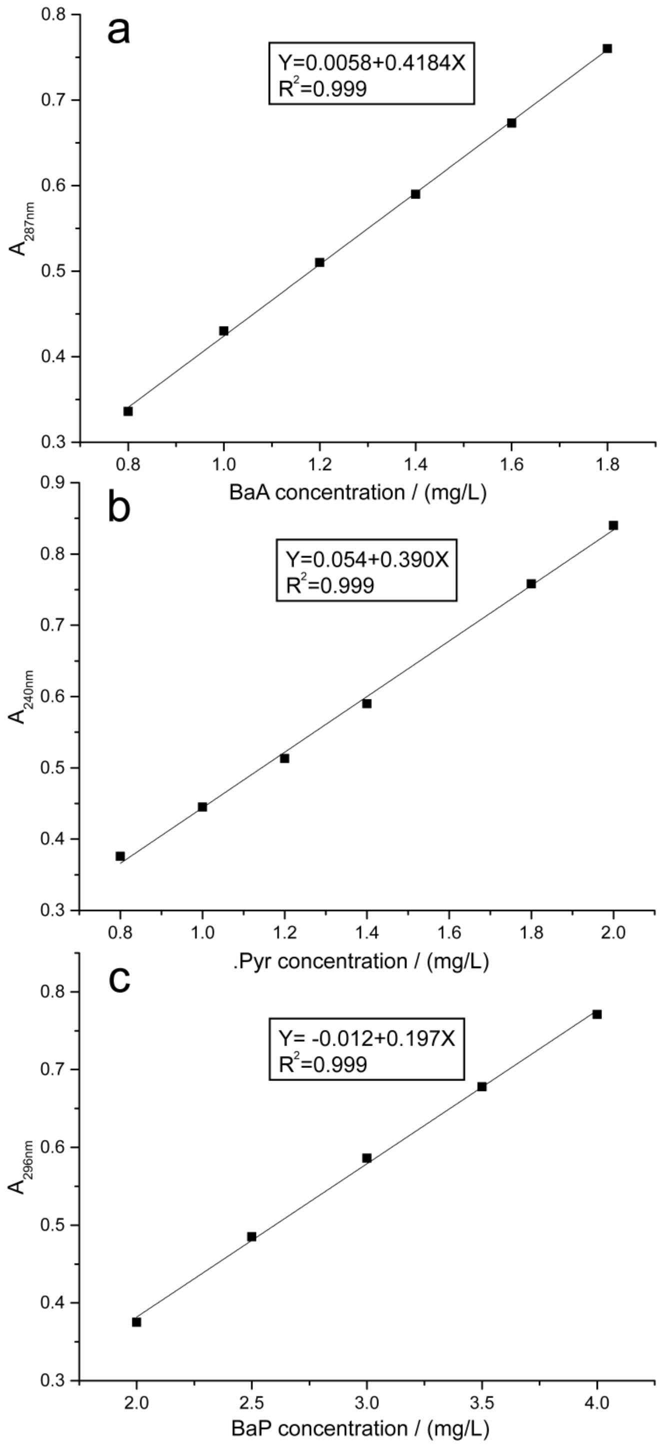

Figure S1. The standard curve of Benzo (a) anthracene (a), Pyrene (b) and Benzo (a) pyrene (c) concentration

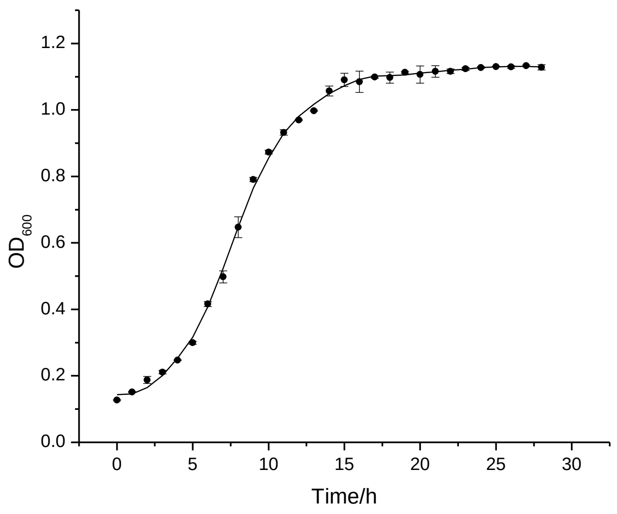

Figure S2. The growth curve of the bacteria

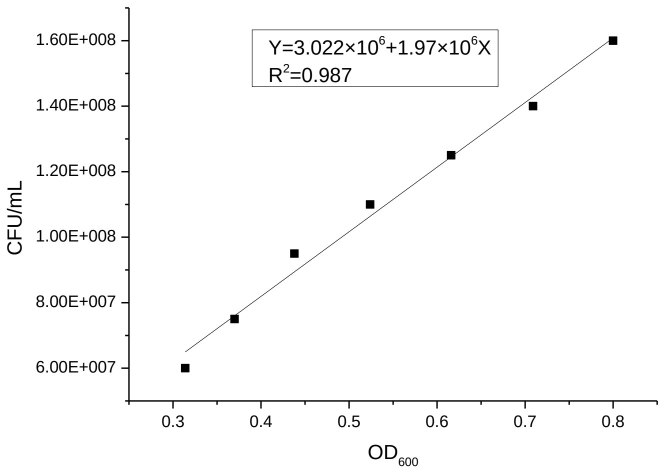

Figure S3. The standard curve of the number of bacteria and the absorbance value

Table S1. Morphological, physiological and biochemical properties of bacteria from oil-contaminated sludge.

Property	PAH-2
Colony color	Red
Colony surface	No wet or smooth
Shape of cells	Short rod
Gram staining	+
Spore staining	+
Oxidase test	-
Catalase test	-
Glucose oxidation	Fermented
MR test	-
V-P test	-
Indole test	-
Hydrated cellulose	-

+ represents positive; - represents negative.
